# KCTD11 inhibits progression of lung cancer by binding to β‐catenin to regulate the activity of the Wnt and Hippo pathways

**DOI:** 10.1111/jcmm.16883

**Published:** 2021-08-28

**Authors:** Man Yang, Ya‐mei Han, Qiang Han, Xue‐zhu Rong, Xiao‐fang Liu, Xu‐Yong Ln

**Affiliations:** ^1^ Department of Pathology The First Affiliated Hospital and College of Basic Medical Sciences China Medical University Shenyang China; ^2^ Department of Pathology Zhongshan Hospital Fudan University Shanghai China

**Keywords:** Hippo pathway, KCTD11, Wnt pathway, YAP, β‐catenin

## Abstract

KCTD11 has been reported to be a potential tumour suppressor in several tumour types. However, the expression of KCTD11 and its role has not been reported in human non‐small cell lung cancer (NSCLC). Whether its potential molecular mechanism is related to its BTB domain is also unknown. The expression of KCTD11 in 139 NSCLC tissue samples was detected by immunohistochemistry, and its correlation with clinicopathological factors was analysed. The effect of KCTD11 on the biological behaviour of lung cancer cells was verified in vitro and in vivo. Its effect on the epithelial‐mesenchymal transition(EMT)process and the Wnt/β‐catenin and Hippo/YAP pathways were observed by Western blot, dual‐luciferase assay, RT‐qPCR, immunofluorescence and immunoprecipitation. KCTD11 is under‐expressed in lung cancer tissues and cells and was negatively correlated with the degree of differentiation, tumour‐node‐metastasis (TNM) stage and lymph node metastasis. Low KCTD11 expression was associated with poor prognosis. KCTD11 overexpression inhibited the proliferation and migration of lung cancer cells. Further studies indicated that KCTD11 inhibited the Wnt pathway, activated the Hippo pathway and inhibited EMT processes by inhibiting the nuclear translocation of β‐catenin and YAP. KCTD11 lost its stimulatory effect on the Hippo pathway after knock down of β‐catenin. These findings confirm that KCTD11 inhibits β‐catenin and YAP nuclear translocation as well as the malignant phenotype of lung cancer cells by interacting with β‐catenin. This provides an important experimental basis for the interaction between KCTD11, β‐catenin and YAP, further revealing the link between the Wnt and Hippo pathways.

## 1 INTRODUCTION

The highly conserved Hippo pathway, first identified in Drosophila,^1^ is involved in regulating the biological behaviour of cells as well as the size of tissues and organs.^2^, ^3^ Abnormalities in this pathway were highly related to the occurrence and metastasis of various cancers.^4^ When the Hippo pathway is inactivated, YAP and TAZ are dephosphorylated, allowing them to translocate into the nucleus and interact with transcription factors (such as TEADs) to enhance the transcriptional activity of downstream effector molecules and promote cell proliferation.^5^, ^6^, ^7^ Wnt signalling pathway is also involved in many biological processes, including tissue and organ growth, cell proliferation, apoptosis and stem cell maintenance.^8^, ^9^, ^10^ β‐catenin acts as a transcriptional activator of the canonical Wnt signalling pathway. When the canonical Wnt pathway is stimulated by Wnt signalling, β‐catenin cannot be degraded by a complex composed of GSK‐3β, Axin and APC; thus, its nuclear translocation is increased, allowing it to interact with the transcription factor TCF/LEF to promote the transcriptional activity of downstream target genes.^11^, ^12^

Recent studies have shown that there is a complex crosstalk between the Hippo / YAP and Wnt/β‐catenin signalling pathways. Specifically, YAP/TAZ combined with β‐catenin to block β‐catenin nuclear localization is the core mechanism of how the Hippo pathway inhibits the Wnt pathway. ^13^, ^14^ Park et al. found that YAP/TAZ, as a downstream effector of the Wnt signalling pathway, can be induced and activated by Wnt5a/b and Wnt3a, which enhance TEAD‐mediated transcription activities.^15^ Azzolin et al. found that APC directly regulated the degradation of YAP/TAZ through the β‐catenin degradation complex.^16^ Studies by Konsavage et al. showed that knockdown of β‐catenin reduces YAP mRNA and protein levels.^8^ Elucidating the mechanisms of mutual regulation of these two pathways may reveal new directions for the development of tumour‐targeted drugs.

KCTD11 is a member of the potassium channel tetramerization domain (KCTD) family. Studies have shown that the highly conserved BTB domain (Bric‐a‐brac/Tramtrack/Broad complex) is involved in complex intracellular signalling. ^17^, ^18^ Cullin 3 (Cul3), a scaffold protein involved in the degradation of a variety of intracellular proteins, is a major regulator of the cell cycle and developmental processes. ^19^, ^20^ The N‐terminal BTB domains of some KCTD proteins can be used as bridges connecting Cul3 and substrates and play the role of Cul3 ubiquitin ligase. They have multiple biological functions and are closely related to protein ubiquitination.^21^, ^22^ However, whether KCTD11 can promote the degradation of Hippo/YAP and Wnt/β‐catenin and the mechanism by which it may exert tumour inhibition is unknown. In this study, we first verified the level of KCTD11 expression in lung cancer tissues and cells. We also investigated the effects of KCTD11 on the proliferation and invasion of lung cancer cells. We demonstrated that KCTD11 inhibits β‐catenin expression and directly binds to β‐catenin. Therefore, we aimed to investigate whether KCTD11 can regulate the Wnt pathway by binding to β‐catenin via the BTB domain, thereby regulating the Hippo pathway, as well as clarify the role of this complex in crosstalk between the Hippo and Wnt signalling pathways.

## 2 MATERIALS AND METHODS

### 2.1 Patients and specimens

After obtaining informed consent from the local medical examination committee of China Medical University, tissue samples from 139 patients with a diagnosis of non‐small cell lung cancer (78 males and 61 females) were obtained from those who underwent surgical resection at the First Affiliated Hospital of China Medical University from 2014 to 2017. The average age of the patients was 60 years. None of the patients underwent either radiotherapy or chemotherapy before surgical resection and received standard chemotherapy after surgery. According to the 2015 World Health Organization classification guidelines for lung tumours,^23^ immunohistochemical staining was used to assess histological type and degree of differentiation. The sample comprised 68 cases of squamous cell carcinoma and 71 cases of adenocarcinoma, of which 79 cases were classified as highly differentiated and 60 cases were classified as moderately or poorly differentiated. According to the pathological tumour lymph node metastasis (TNM) staging of the International Union against Cancer (seventh edition) (Detterbeck et al., 2017), specimens can be classified into stages I–II (*n* = 73) and III (*n* = 66). Lymph node metastases occurred in 62 of 139 patients. In addition, a total of 20 newly isolated specimens (including tumours and normal tissues) were collected from surgical resection and immediately stored at 80℃ to extract tissue proteins.

### 2.2 Immunohistochemistry (IHC)

The analysis was performed as previously described.^24^ Briefly, tissue sections were cultured with KCTD11 rabbit polyclonal antibody (1:100 dilution; Sigma‐Aldrich). Two independently blinded investigators examined all tumour sections by taking five random fields from each section, with 100 cells in the field of view observed and magnified 400 times for scoring. Due to differences in the lesions, the proportion of positive cells and staining intensity were considered. The KCTD11 staining positive cell rate was assigned as follows: 1 (1%–25%), 2 (26%–50%), 3 (51%–75%) and 4 (76%–100%). The staining intensity was divided into 0 (no staining), 1 (weak staining, light yellow staining), 2 (medium staining, yellow staining) or 3 (strong staining, brown staining). The two scores for each tumour sample were multiplied to give a final score of 0–12, with a tumour sample score ≥4 defined as positive expression, scores of 1–4 defined as low expression and a score of 0 defined as negative expression. Phosphate buffer (MaiXin) and goat serum (MaiXin) were used as negative controls.

### 2.3 Cell culture

The HBE(human bronchial epithelioid cells)cell line was obtained from the American Type Culture Collection (ATCC); (Manassas, VA, USA). The LK2 cell line was a gift from H. Kijima (Department of Pathology and Bioscience, Hirosaki University Graduate School of Medicine, Japan). Other NSCLC cell lines, including A549, SPC, H1299, H292, H460 and H661, were purchased from the Shanghai Cell Bank (Shanghai, China). Under conditions of high humidity (37℃ and 5% CO_2_), all cells were cultured in RPMI 1640 (Invitrogen, Carlsbad, CA) supplemented with 10% foetal bovine serum (Gibco), 100 U/ml penicillin and 100 μg/ml streptomycin (Sigma‐Aldrich). All cell lines were identified using short tandem repeat DNA analysis.

### 2.4 Plasmid construction and transfection

Plasmids pCMV6‐Myc‐DDK (empty vector, EV) and pCMV6‐Myc‐DDK‐tagged‐KCTD11 were purchased from Origene. Small interfering RNAs for KCTD11 (siKCTD11; sc‐93577) and non‐targeting siRNAs (control siRNA, NC; sc‐36869) were purchased from Santa Cruz Biotechnology Inc. siRNAs for CTNNBIP1 (si‐β‐catenin) and non‐targeting siRNAs (siNC) were purchased from General Biosystems Inc. The MYC‐KCTD11‐ΔBTB plasmid was constructed by RiboBio (Guangzhou, China). Transfection was performed using Lipofectamine 3000 (Thermo Fisher Scientific), according to the manufacturer's instructions. Stable clonal cell lines were constructed using G418.

### 2.5 Western blot analysis and co‐immunoprecipitation

Total protein samples were extracted quantitatively from the lysis buffer (Pierce, Rockford, IL). The reagent or bound protein was eluted from protein G PLUS‐agarose (Santa Cruz Biotechnology), separated by SDS‐PAGE, transferred onto polyvinylidene fluoride membranes (PVDF; Millipore, Billerica, MA, USA), incubated with antibodies overnight at 4℃, followed by addition of oxidase‐conjugated anti‐mouse or anti‐rabbit IgG (Santa Cruz Biotechnology) at 37℃ for washing and incubation for 2 h. Target protein expression levels were detected using an ECL kit (Thermo Fisher Scientific) and a bioimaging system (Bio‐Rad). WB analysis and IP were performed with the following antibodies: Myc‐tag, LATS1, YAP, P‐YAP, CTGF, β‐catenin, C‐myc, CyclinD1, MMP7, ZO‐1, N‐cadherin, E‐cadherin, vimentin, Snail, Slug, active β‐catenin, P‐β‐catenin (Cell Signaling Technology Inc. 1 μg/IP, 1:1000/WB); GAPDH (Sigma, 1:1000/WB); KCTD11 (Sigma, 1 μg/IP,1:1000/WB); LaminB1 (Abcam, 1:1,000/WB); and Tubulin (Abcam,1:1,000/WB).

### 2.6 Colony formation, Transwell and MTT assays

After 24 h of transfection with plasmids or siRNA, the cells were seeded in a 6 cm cell culture dish (1000 cells per dish) and incubated for 10 days. When the number of single colony cells reached approximately 50, cells were washed with PBS, fixed with 4% paraformaldehyde and stained with crystal violet, and the number of colonies was statistically analysed.

#### 2.6.1 Transwell migration assay

A cell migration assay was performed using a 24‐well Transwell chamber with an 8‐pore size (Costar). After 24 h of transfection, the cells were counted by trypsinization, 100 μl of serum‐free medium containing 3 × 10^5^ cells was evenly spread to the upper chamber while a medium containing 10% FBS as a chemical attractant was added to the lower chamber, after which the samples were cultured for 16 h. In the 4% paraformaldehyde‐fixed cells, after clearing the non‐migrating cells in the upper chamber using a cotton swab, crystal violet staining was performed. The number of migrated cells was counted under a microscope, and ten high‐power fields were randomly selected.

#### 2.6.2 MTT assays

Cell counts were measured 24 h after transfection, and cells were plated in 96‐well plates in media containing 10% FBS at approximately 3,000 cells/well. Cell viability was determined after five consecutive days. Briefly, 20 μl of 5 mg/ml MTS solution was added to each well in the dark and cultured for 2 h, and the results were spectrophotometrically determined using a test wavelength of 490 nm.

### 2.7 Immunofluorescence staining

The cells were fixed with 4% polyoxymethylene, permeabilized with Triton X‐100, blocked with normal goat serum at 37℃ for 1 h and incubated with primary antibody (KCTD11: 1:100, Sigma, β‐catenin, YAP: 1:100, CST) overnight at 4℃. Next, they were incubated with fluorescein isothiocyanate‐conjugated (FITC) or tetramethylrhodamine isothiocyanate‐conjugated (TRITC) secondary antibody at 37℃ for 2 h. Cell nuclei were stained with 4,6‐diamidino‐2‐phenylindole (DAPI). Cell images were acquired and analysed using an inverted Nikon TE300 microscope (Nikon Co., Ltd.) or a radiant 2,000 laser scanning confocal microscope (Oberkosen Carl Zeiss).

### 2.8 Dual‐luciferase assay

Luciferase activity in cell extracts was determined using a dual‐luciferase reporter assay kit (Promega). The reporter activity was normalized to co‐express β‐galactosidase activity. All luciferase plasmids were purchased from Addgene. The transcriptional activity of YAP/TEAD in the Hippo pathway was determined using the pGL3b_8xGTIIC‐luciferase plasmid (plasmid #34615). The transcriptional activity of β‐catenin/HTCF‐4 in the Wnt pathway was detected using the luciferase plasmid M50 Super8x TOPFlash located upstream of the minimal c‐fos promoter driving luciferase expression. Recombinant human Wnt3a (#5036‐WN; R&D Systems, Minneapolis, MN, USA) was added to PBS containing 0.1% bovine serum albumin at a concentration of 10 μg/ml and used in experiments at 50 ng/ml.

### 2.9 RNA extraction and real‐time RT‐PCR (RT‐qPCR)

Twenty‐four hours after cell transfection, RNA was extracted and subjected to RT‐qPCR analysis as described previously (Imajo et al., 2012). The relative transcription levels of the genes were normalized to GAPDH mRNA levels. The relative level of gene expression was expressed as ΔCt = Ct gene‐Ct reference, and the 2‐ΔΔCt method was used to calculate the fold change in gene expression. Primer sequences were as follows (Sangon Biotech, Shanghai, China):

CYR61: 5′‐CTCGCCTTAGTCGTCACCC‐3′ (forward).

5′‐CGCCGAAGTTGCATTCCAG‐3′ (reverse).

CTGF: 5′‐AACTGCAACCTCTCGCACTG‐3′ (forward).

5′‐GCTCGGGCTCCTTGTAATTCT‐3′ (reverse).

Cyclin E:5′‐AGCCAGCCTTGGGACAATAAT‐3′ (forward).

5′‐GAGCCTCTGGATGGTGCAAT‐3′ (reverse).

MMP7:5′‐GTTGTATGGGGAACTGCTGAC‐3′ (forward).

5′‐GTCCAGCGTTCATCCTCAATC‐3′ (reverse).

Axin2:5′‐TGACGGACAGCAGTGTAGATG‐3′ (forward).

5′‐GGTTCTCGGGAAATGAGGTAG‐3′ (reverse).

Cyclin D1:5′‐CCAATAGCAGTGCGGAGTCT‐3′ (forward).

5′‐TCAAGCTGAGGGGTGGAGTT‐3′ (reverse).

GAPDH: 5′‐GGAGCGAGATCCCTCCAAAAT‐3′ (forward).

5′‐GGCTGTTGTCATACTTCTCATGG‐3′ (reverse).

### 2.10 Transplantation of tumour cells into nude mice

After approval by the Animal Research Committee of China Medical University and in order to comply with experimental animal ethics guidelines issued by China Medical University, nude mice were used in this study. Four large female BALB/c nude mice were purchased from Charles River. Tumour cells were suspended in 0.2 ml of sterile phosphate‐buffered saline (PBS), and each mouse was inoculated subcutaneously with 5 × 10^6^ tumour cells or subcutaneously inoculated with 2 × 10^6^ tumour cells (KCTD11 transfected A549). Six weeks after inoculation, the mice were sacrificed and an autopsy was performed to examine the growth and spread of the tumour. The excised tumour tissue was fixed in 4% formaldehyde (Sigma) and embedded in paraffin. After H&E staining, tumour clusters in the lung tissue were analysed under a microscope.

### 2.11 Statistical analysis

Statistical analysis was performed using the statistical software SPSS 22.0, which was used to assess the correlation between KCTD11 expression and clinicopathological factors. Prognostic value was tested using a Cox regression model. All clinical pathology parameters were included in the Cox regression model and were tested by univariate analysis using the enter method and by multivariate analysis using a forward stepwise logistic regression method. Image J was used for image analysis of the Western blot results. Differences between test groups were compared using a paired t test using GraphPad Prism software. Differences were considered statistically significant at *p* < 0.05.

## 3 RESULTS

### 3.1 KCTD11 was downregulated in NSCLC tissues and cells

**FIGURE 1:**
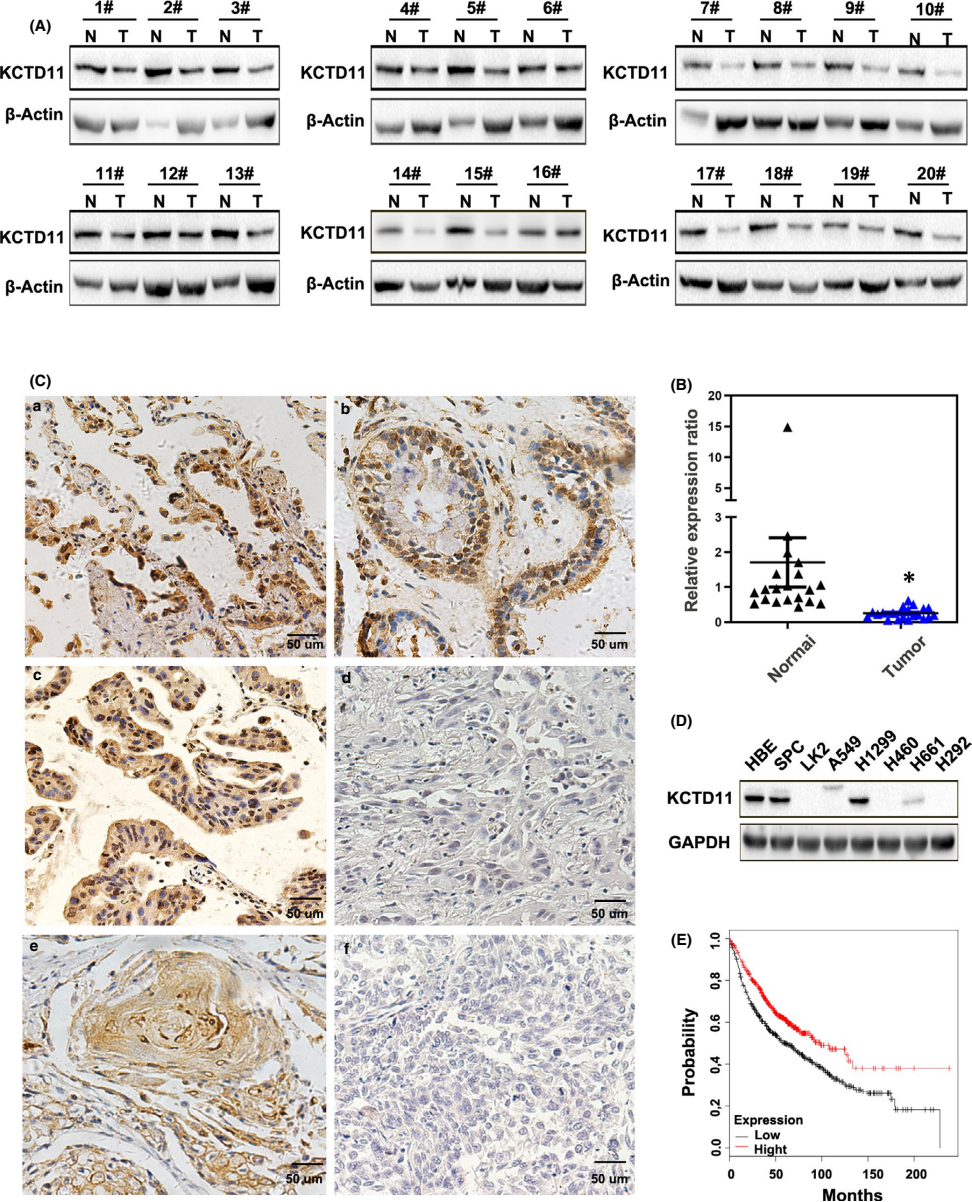
Low expression of KCTD11 in lung cancer is associated with poor prognosis. (A, B) The expression levels of KCTD11 are downregulated in 20 cases of NSCLC tissues compared to normal tissues, as assessed by Western blot (A) and analysed with GraphPad prism (B). (C) Immunohistochemical staining of KCTD11 in the surrounding normal tissues and the representative carcinoma (*n* = 139). KCTD11 is expressed in the nuclei and cytoplasm and show strong positive expression in normal alveolar cells (a) and bronchial epithelial cells (b); positive expression of KCTD11 in highly differentiated adenocarcinoma (c) and squamous cell carcinoma (e); negative expression of KCTD11 in poorly differentiated adenocarcinoma (d) and squamous cell carcinoma (f) is observed (Magnification:×200). (D) Western blot is used to detect the protein level of KCTD11 in eight lung cell lines. (E) The data from Kaplan‐Meier database show that the survival time of KCTD11‐positive patients is significantly longer than that of KCTD11‐negative patients (*p*  <  0.05). GAPDH and β‐Actin serve as the loading control

We examined the expression of KCTD11 in NSCLC tissues and cells. Western blotting showed that the expression of KCTD11 was significantly higher in 20 cases of normal tissues than in paired NSCLC tissues (Figure 1A, B). Immunohistochemical staining also showed that KCTD11 was negatively expressed in 54.7% of NSCLC tissues (76/139, Table 1). KCTD11 was weakly expressed in the nuclei and cytoplasm or negatively expressed in NSCLC tissues, in contrast to its strong expression in normal tissues (Figure 1C). The protein expression of KCTD11 was also higher in HBE cells than in NSCLC cell lines (H1299, LK2, A549, H661, H1299 and H292) (Figure 1D).

**TABLE 1:** The relationship between KCTD11expression with clinicopathological factors in 139 cases of NSCLC

Clinicopathological factors	*N*	Positive	Negative	X2	*p* value
Age(years)					
<60	65	29	36	0.025	0.875
≧60	74	34	40		
Gender					
Male	78	37	41	0.320	0.572
Female	61	26	35		
Histological type					
Squamous cell carcinoma	68	26	42	2.699	0.100
Adenocarcinoma	71	37	34		
Differentiation					
Well	79	42	37	4.540	0.033
Moderate and Poor	60	21	39		
TNM					
Ⅰ+Ⅱ	73	39	34	4.071	0.044
Ⅲ	66	24	42		
Lymph node metastasis					
Positive	62	21	41	5.924	0.015
Negative	77	42	35		

### 3.2 Expression of KCTD11 correlated with clinical factors

We investigated the relationship between KCTD11 expression and clinicopathological factors in patients with NSCLC. The low KCTD11 expression was significantly correlated with the degree of differentiation (*p *= 0.033), advanced pathological TNM (pTNM) stage (*p* = 0.044) and positive lymph node metastasis (*p *= 0.015), but not with age, sex and histological type (Table 1). Moreover, Kaplan‐Meier survival analysis showed that the overall survival rate of the KCTD11‐positive group was significantly higher than that of the KCTD11‐negative group (*p* < 0.001, log‐rank test), suggesting an association between KCTD11 expression and prognosis (Figure 1E). Univariate analysis showed that KCTD11, high TNM classification and positive lymph node metastasis were significant prognostic factors for NSCLC (low KCTD11 expression: hazard ratio 0.368, *p *< 0.01; positive lymph node metastasis: hazard ratio 2.611, *p* < 0.001; and high TNM classification: hazard ratio 2.172, *p *= 0.01). Multivariate analysis using a Cox regression model also indicated that low KCTD11 expression and positive lymph node metastasis were independent prognostic factors in patients with NSCLC (Table 2).

**TABLE 2:** Univariate analysis and multivariate analysis in 139 cases of NSCLC

Clinicopathological characteristics	Hazard ratio (95% CI)	*p value*
Univariate analysis		
Age older than 60	1.182 (0.758–1.844)	0.460
Gender: male	0.761 (0.485–1.192)	0.232
Histological type:Adenocarcinoma	0.861 (0.554–1.340)	0.508
Moderate and Poor differentiation	1.515 (0.974–2.356)	0.065
High TNM classification	2.172 (1.376–3.428)	0.001
Positive lymph node metastasis	2.611 (1.636–4.168)	<0.001
Low KCTD11 expression	0.368 (0.226–0.599)	<0.001
Mulivariate analysis		
Positive lymph node metastasis	2.059 (1.098–3.861)	0.024
Low KCTD11 expression	0.401 (0.239 −0.673)	0.001

### 3.3 KCTD11 inhibited proliferation and invasion of HBE and NSCLC cells in vitro and in vivo

**FIGURE 2:**
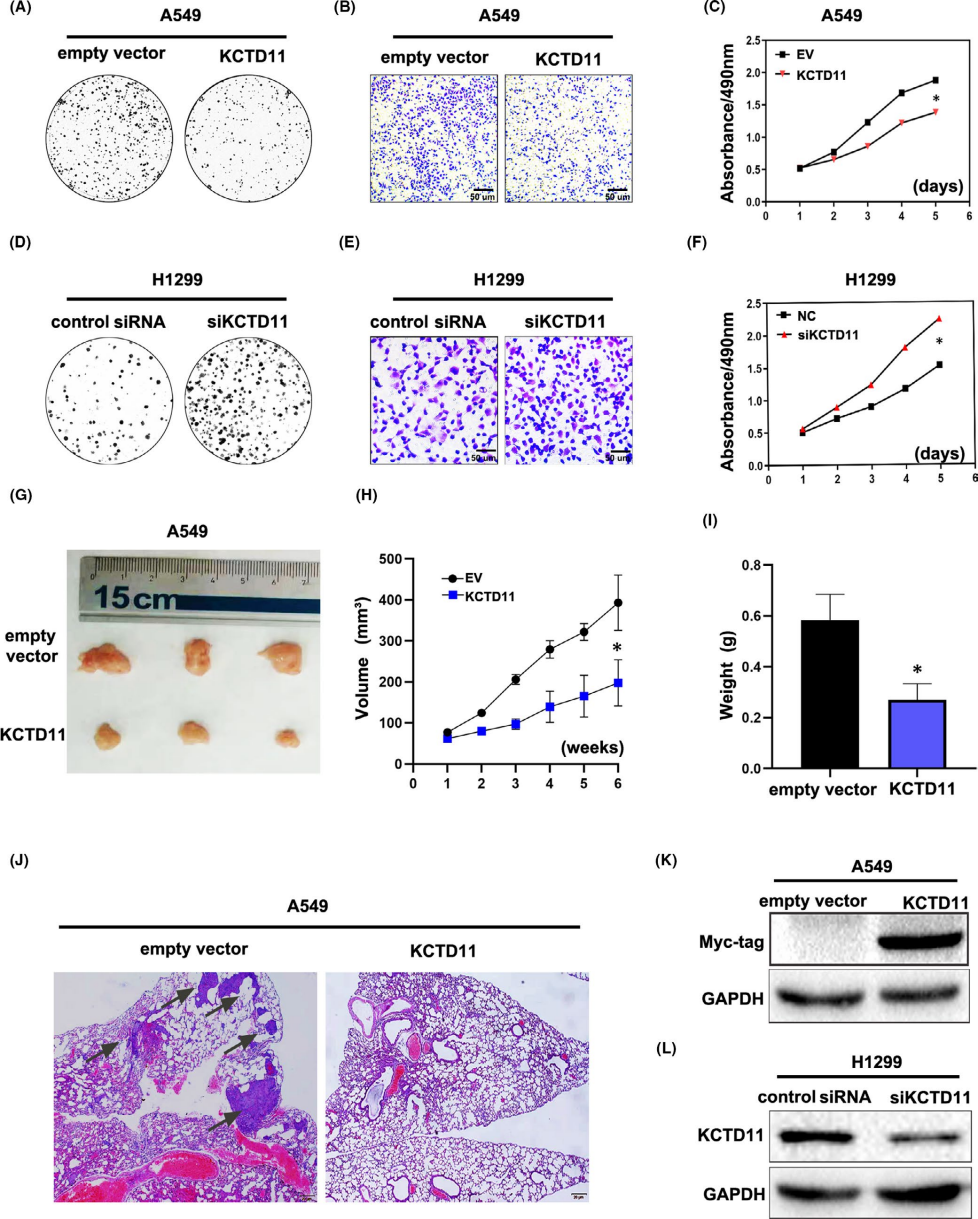
KCTD11 inhibits proliferation and invasion of NSCLC. (A–C) In KCTD11‐overexpressing A549 cells, a colony formation assay, Transwell assay and MTT assay show that the proliferation and invasion of cells are reduced, (D–F) whereas the proliferation and invasion of cells are increased in KCTD11‐depleted H1299 cells. (G‐I) KCTD11 overexpression inhibits tumour formation and metastases in nude mice. Subcutaneous injection of KCTD11‐overexpressing A549 cells (G418 screening) into nude mice (*n* = 3) and the control group (*n* = 3). The mice are sacrificed, and an autopsy performed to examine the growth and spread of the tumour after six weeks. Compared with the control group, the tumour formation is reduced. (J) Mice injected with KCTD11‐overexpressing A549 cells through tail vein have decreased pulmonary metastases than those in the control group, *p  <  0.05. (K‐L) The transfection efficiency is shown by Western blot after overexpressing or interfering KCTD11. All the experiments are repeated three times independently, and the results are the mean. Abbreviations: EV, Empty vector; NC, Negative control

We transfected exogenous expression vectors to overexpress KCTD11 in A549 and H460 cells, and used RNA interference to deplete endogenous KCTD11 in H1299 cells and HBE cells. Transfection of KCTD11 significantly reduced colony formation in A549 cells (KCTD11 vs. control: 162.0 ± 7.000 vs. 72.33 ± 4.978, *p* < 0.001) and H460 cells (KCTD11 vs. control: 166.0 ± 21.59 vs. 99.67 ± 8.253, *p* < 0.05). Conversely, depleting endogenous KCTD11 significantly promoted colony formation in H1299 cells (negative siRNA vs. siRNA‐KCTD11: 90.00 ± 11.59, vs. 212.7 ± 14.45, *p* < 0.05) and HBE cells (negative siRNA vs. siRNA‐KCTD11: 41.67 ± 4.842 vs. 88.00 ± 5.775) (Figure 2A and D; Figure S1A and D). In the Transwell assay, the invasive ability of KCTD11‐overexpressing cells was significantly reduced compared with that of the control group in A549 cells (KCTD11 vs. control: 406.3 ± 11.05 vs. 203.3 ± 21.06, *p* < 0.05) and H460 cells (KCTD11 vs. control: 433.3 ± 57.00 vs. 203.7 ± 49.36, *p* < 0.05). In contrast, depleting endogenous KCTD11 significantly reduced the invasive ability of H1299 cells (negative siRNA vs. si‐KCTD11: 139.0 ± 29.54 vs. 263.3 ± 25.91, *p* < 0.001) and HBE cells (negative siRNA vs. si‐KCTD11: 93.67 ± 2.186 vs. 200.0 ± 11,15, *p* < 0.05) (Figure 2B and E; Figure S1B and E). The MTT assay showed that the proliferation rate of the A549 and H460 cell lines after overexpression of KCTD11 was significantly lower than that of the control group *(p* < 0.05). Consistently, the proliferation rate was significantly reduced in KCTD11‐depleted H1299 cells and HBE cells (*p* < 0.05). (Figure 2C and F; Figure S1C and F).

We then examined the effects of KCTD11 on the growth and metastasis of NSCLC cells *in vivo* using a nude mouse xenograft NSCLC model. A549 cells stably expressing KCTD11 were subcutaneously injected into nude mice. Six weeks later, KCTD11‐overexpressing A549 cells showed less progressive tumour growth in the nude mice than in the control group (KCTD11 vs. control group: 0.5836 ± 0.05839 vs. 0.2707 ± 0.03628, *p* < 0.05, *n* = 3) (Figure 2G, H and I; Figure S1G), whereas KCTD11‐overexpressing A549 cells showed less metastasis in the nude mice than in the control group (Figure 2J and Figure S1H). The KCTD11 protein levels are shown in Figure 2K and L. Collectively, these results indicate that KCTD11 regulates the proliferation and invasion of NSCLC cells in vivo.

### 3.4 KCTD11 activated the Hippo pathway by upregulating the phosphorylation level of YAP

**FIGURE 3:**
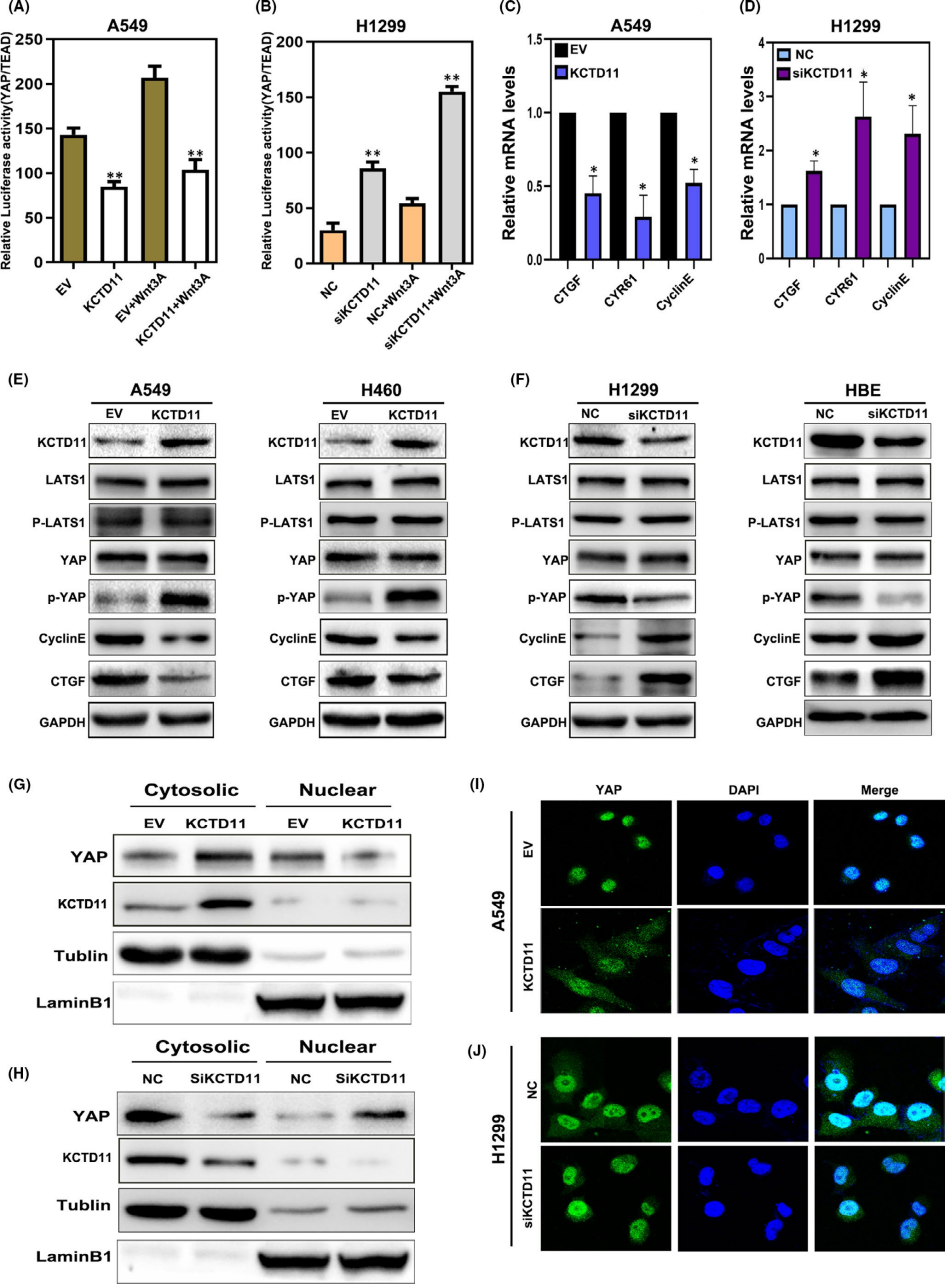
KCTD11 activates the Hippo pathway by upregulating the phosphorylation level of YAP in NSCLC cells. (A, B) KCTD11 overexpression inhibits the transcriptional activity of TEAD. By Hippo pGTII luciferase reporter, transfection of KCTD11 in A549 cells upregulates the activity of Hippo signalling pathway, and depleted KCTD11 in H1299 cells inhibits the activity of Hippo signalling pathway. The experiment is treated with control or wnt3a conditioned medium for 6 h. (C, D) The target mRNA expression levels of the Hippo pathway, CTGF, CYR61 and CyclinE are downregulated in KCTD11‐overexpressing A549 cells and are upregulated in KCTD11‐depleted H1299 cells. (E, F) Western blot shows that the phosphorylation of YAP is increased, and expression levels of target genes of the Hippo pathway, CTGF and CyclinE are downregulated in KCTD11‐overexpressing A549 cells and H460 cells. In KCTD11‐depleted H1299 cells and HBE cells, the phosphorylation of YAP decreases, and expression levels of CTGF and CyclinE are upregulated. GAPDH is used as a loading control. The grey value is analysed by using Image J software. (G–I) Nucleus‐cytoplasm isolation and immunofluorescence demonstrate that the nuclear translocation of YAP is inhibited in A549 cell lines after transfection of KCTD11 and silencing of KCTD11 in H1299 cells leads to an increase in the nuclear translocation of YAP. (I, J, magnification 400×; Bars: 20 µm). Results are shown from three independent experiments

The dual‐luciferase assay showed that transfection of KCTD11 upregulated the activity of the Hippo signalling pathway, and that depletion of KCTD11 inhibited the activity of the Hippo signalling pathway (Figure 3A, B). We then found that CTGF, CYR61 and cyclin E mRNA, which are related to the Hippo signalling pathway, were downregulated in KCTD11‐overexpressing A549 cells and upregulated in KCTD11‐depleted H1299 cells (Figure 3C, D), suggesting that KCTD11 is involved in the Hippo signalling pathway. Therefore, we investigated whether KCTD11 is involved in YAP/P‐YAP during Hippo signal transduction. No significant changes in YAP and LATS1 were found in A549 and H460 cell lines overexpressing KCTD11, as well as in H1299 and HBE cell lines lacking endogenous KCTD11. However, we found that P‐YAP was upregulated by KCTD11 overexpression. When endogenous KCTD11 was depleted, P‐YAP was consistently downregulated in H1299 and HBE cells. Cyclin E and CTGF, which are related to proliferation, were downregulated when KCTD11 was overexpressed and upregulated in the absence of endogenous KCTD11 expression (Figure 3E, F). We also found that nuclear translocation of YAP was inhibited in A549 cell lines after transfection with KCTD11, which can be demonstrated by nucleus‐cytoplasm isolation and immunofluorescence (Figure 3G and I). These results were reversed in the H1299 cell line, with depletion of KCTD11 promoting the nuclear translocation of YAP (Figure 3H and J). Therefore, these results indicate that KCTD11 activates the Hippo signalling pathway by promoting the phosphorylation of YAP.

### 3.5 KCTD11 inhibits Wnt pathway and nuclear translocation of β‐catenin

**FIGURE 4:**
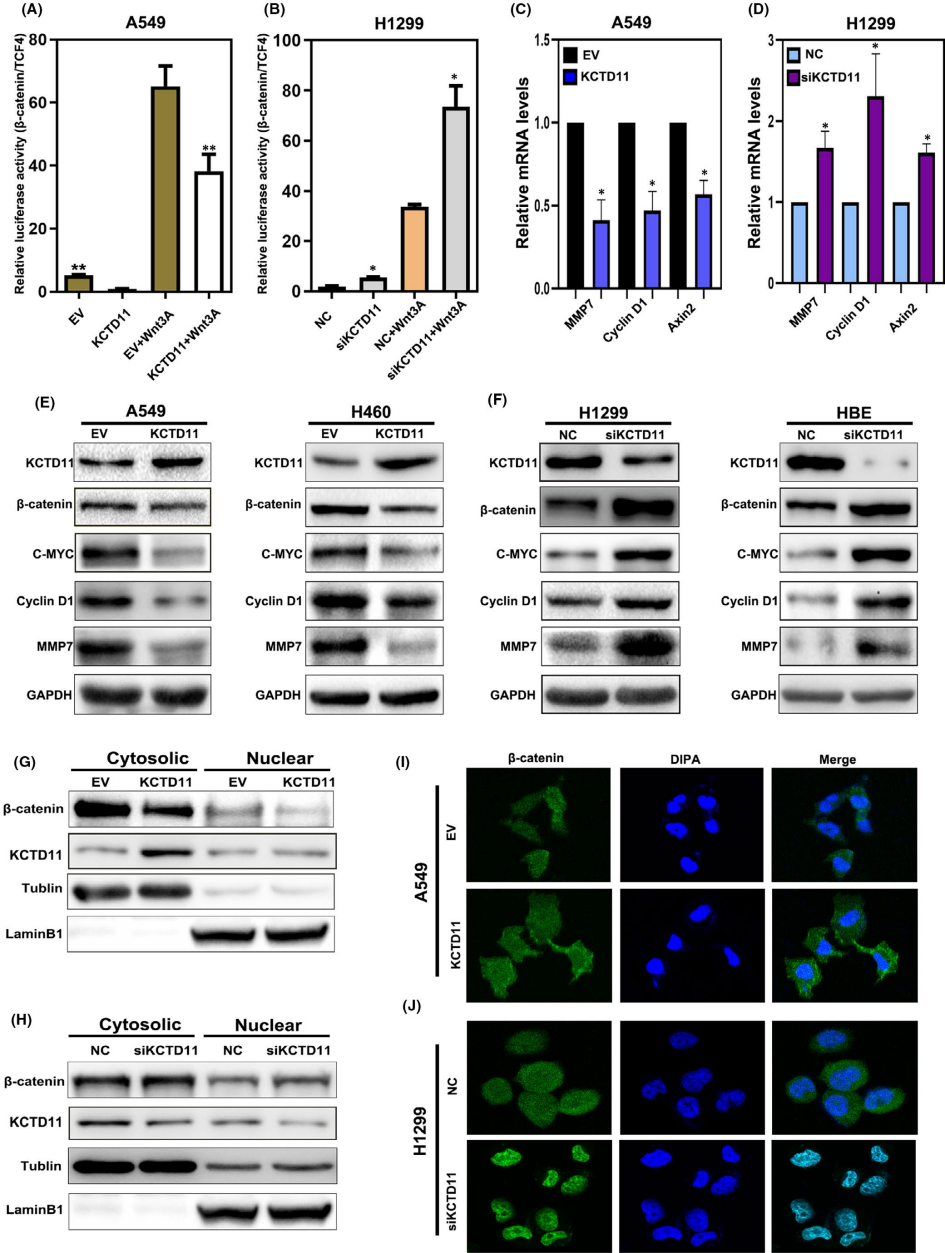
KCTD11 inhibits the Wnt pathway and nuclear translocation of β‐catenin. (A, B) By Wnt TOPflash reporter, transfection of KCTD11 in A549 cells downregulates the activity of Wnt signalling pathway, and depleted KCTD11 in H1299 cells upregulates the activity of Wnt signalling pathway. The experiment is treated with control or wnt3a conditioned medium for 6 h. (C, D) The target genes mRNA expression levels of the Wnt pathway, MMP7, CyclinD1 and Axin2 are downregulated in KCTD11‐overexpressing A549 cells and are upregulated in KCTD11‐depleted H1299 cells. (E, F) Western blot shows that the levels of β‐catenin and the transcriptional activity of Wnt pathway downstream target genes, C‐myc, cyclin D1 and MMP7, are downregulated in KCTD11‐overexpressing A549 cells and H460 cells but upregulated in KCTD11‐depleted H1299 cells and HBE cell. GAPDH is used as a loading control. The grey value is analysed by using Image J software. (G–I) Nucleus‐cytoplasm isolation and immunofluorescence demonstrate that the nuclear translocation of β‐catenin is decreased in A549 cell lines after transfection of KCTD11 and silencing of KCTD11 in H1299 cells leads to an addition in the nuclear translocation of β‐catenin. (I, J, magnification 400×; Bars: 20 µm). Results are shown from three independent experiments

According to previous reports, crosstalk exists between the Wnt and Hippo pathways. Therefore, to further explore the molecular mechanism through which KCTD11 affects the malignant phenotype of lung cancer, we focused on the effects of KCTD11 on the Wnt pathway. To readily observe the differences in Wnt pathway activity by luciferase reporter assays, we stimulated the Wnt signalling pathway with Wnt‐3A and found that the luciferase activity of β‐catenin/TCF‐4 was significantly downregulated in KCTD11‐overexpressing A549 cells but significantly upregulated in KCTD11‐depleted H1299 cells (Figure 4A, B). At the same time, we found that MMP7, cyclin D1 and Axin2 mRNA, which are related to the Wnt signalling pathway, were downregulated in KCTD11‐overexpressing A549 cells and upregulated in KCTD11‐depleted H1299 cells (Figure 4C, D). WB also showed that the levels of β‐catenin, c‐myc, MMP7 and cyclin D1 were downregulated in KCTD11‐overexpressing A549 and H460 cells but upregulated in KCTD11‐depleted H1299 cells and HBE cells. (Figure 4E, F). In A549 cell lines, transfection with KCTD11 inhibited the nuclear translocation of β‐catenin, as demonstrated by nucleus‐cytoplasm isolation and immunofluorescence (Figure 4G and I). In H1299 cells, depletion of KCTD11 promoted the nuclear translocation of β‐catenin (Figure 4H and J). These results indicate that KCTD11 negatively regulates the Wnt signalling pathway by inhibiting the nuclear translocation of β‐catenin.

### 3.6 KCTD11 inhibits the EMT process in non‐small cell lung cancer

**FIGURE 5:**
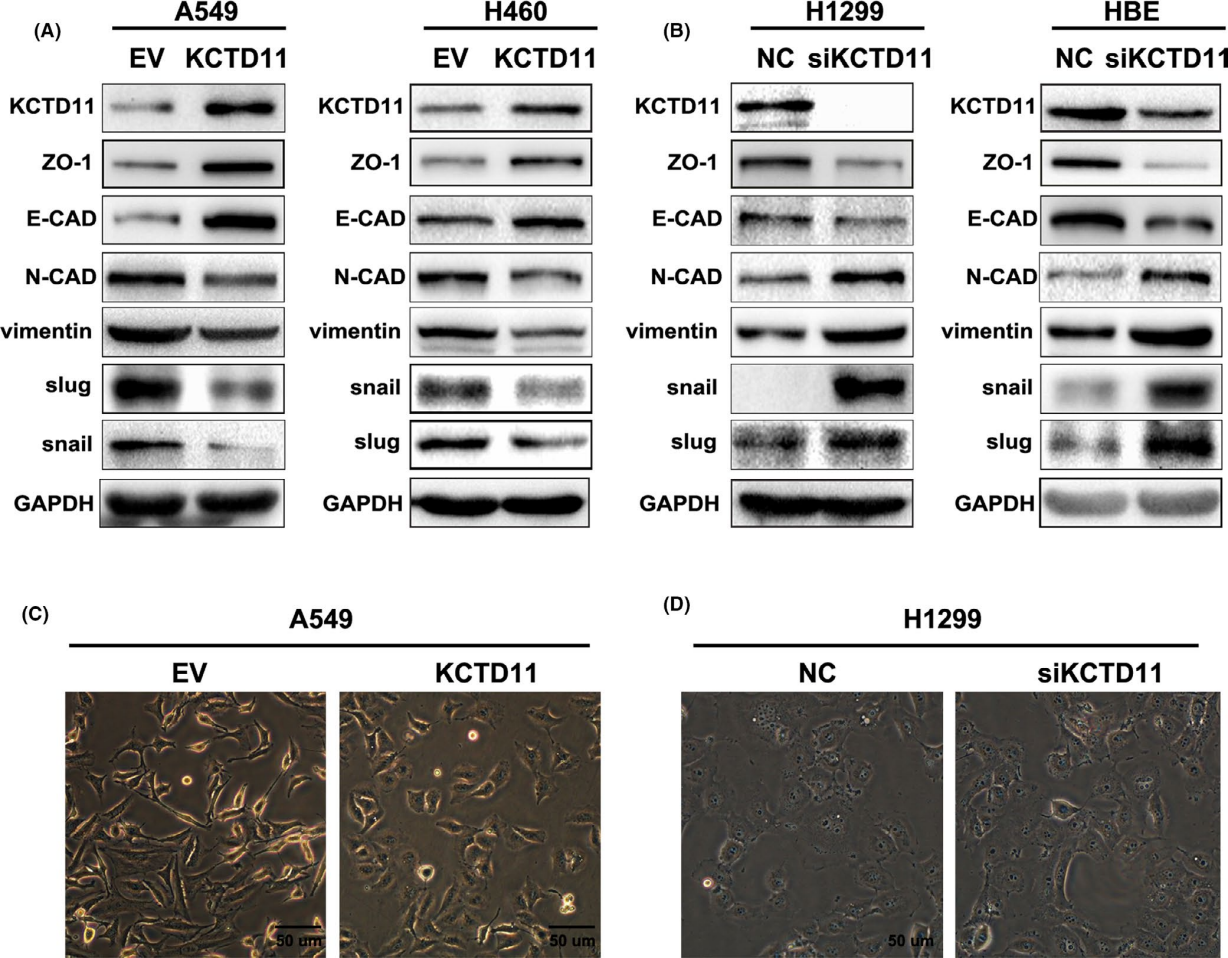
KCTD11 inhibits the process of EMT. (A, B) A549 cells and H460 cells are transfected with KCTD11, the cells are lysed 48 h later, and the level of proteins are detected by Western blot. E‐cadherin and ZO‐1 are upregulated while N‐cadherin, vimentin, Snail and Slug are downregulated in KCTD11‐overexpressing A549 cells and H460 cells, while the results are reversed in KCTD11‐depleted H1299 cells and HBE cells. GAPDH is used as a loading control. (C, D) KCTD11 induces a morphology change and representative phase‐contrast images of cells growing in monolayer cultures. KCTD11 transfection preserves the cobblestone‐like appearance of tight intercellular junctions in A549 cells, while depleting endogenous KCTD11 in H1299 cells show the opposite effect, with the cells exhibiting a spindle‐like morphology (magnification, 200×;Bars: 50 µm)

The EMT process is usually accompanied by a decrease in E‐cadherin, ZO‐1 and occludin, with an accompanying increase in the mesenchymal marker N‐cadherin. Snail and Slug are transcription factors considered as key regulators of EMT. Western blot analysis confirmed that levels of E‐cadherin and ZO‐1 were upregulated in KCTD11‐overexpressing A549 cells and H460 cells but downregulated in KCTD11‐depleted H1299 cells and HBE cells, and that N‐cadherin, Snail and Slug were downregulated in KCTD11‐overexpressing A549 and H460 cells but upregulated in KCTD11‐depleted H1299 cells and HBE cells (Figure 5A, B). At the same time, A549 cells transfected with KCTD11 retained a cobblestone‐like appearance with tight cell‐cell junctions, while the control group showed a spindle‐like morphology with a scattered distribution as an EMT feature. In contrast, depletion of endogenous KCTD11 in H1299 cells displayed a spindle‐like morphology (Figure 5C, D). Therefore, these results indicate that KCTD11 levels are associated with EMT and negatively regulates the transcription factors Snail and Slug.

### 3.7 KCTD11 binds to β‐catenin via the BTB domain

**FIGURE 6:**
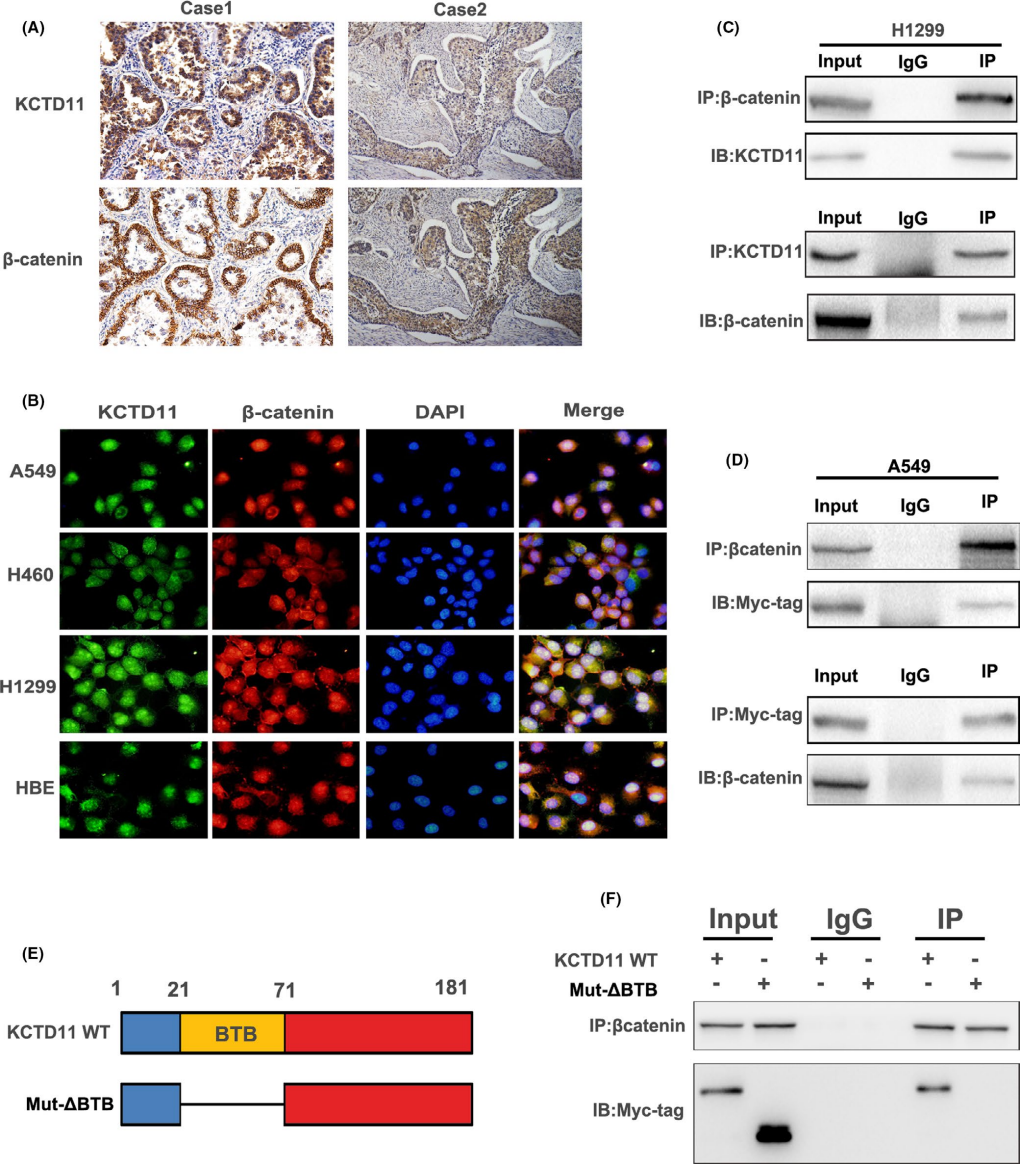
KCTD11 binds to β‐catenin via the BTB domain. (A) Immunohistochemical staining of serial sections and statistical analysis to show that KCTD11 is negatively correlated with β‐catenin expression (*p* = 0.035). (B) Immunofluorescence reveals that endogenous KCTD11 and β‐catenin had co‐localization in NSCLC cell lines. (C) Interaction between endogenous KCTD11 and β‐catenin is detected by co‐immunoprecipitation in A549 cells. Cell lysates are immunoprecipitated with anti‐KCTD11 antibody or anti‐β‐catenin, and then, the expression level of KCTD11 and β‐catenin by immunoblotting is examined. The IgG serves as negative control. (D) The interaction between exogenous KCTD11 and β‐catenin is also detected by immunoprecipitation with transfection of Myc‐tagged KCTD11 plasmids into A549 cells. (E) Different locations within the structure of wild KCTD11 plasmid and Mutant MYC‐KCTD11‐ΔBTB (mut‐ΔBTB) plasmid. (F) KCTD11interacts with β‐catenin via its BTB domain. A549 cells are transfected with KCTD11 and mut‐ΔBTB plasmid, and the resultant cell lysates are immunoprecipitated with anti‐β‐catenin antibody. Deletion of BTB domain preventing KCTD11 from binding to β‐catenin is confirmed by the absence of Myc‐tag. (magnification, 200×;Bars: 50 µm)

Recent studies have shown that the BTB domain of the KCTD family is a highly versatile scaffold that acts as a bridge to substrates involved in the ubiquitination of multiple proteins (Wang et al., 2016). The relationship between KCTD1 and β‐catenin has been demonstrated previously (Li et al., 2014). We speculated that the BTB domain of KCTD11 might interact with β‐catenin. We performed immunohistochemical staining of serial sections and statistical analysis to show that KCTD11 expression was correlated with β‐catenin membranous expression (*p* = 0.035) (Table 3, Figure 6A). Immunofluorescence staining also revealed that the two proteins were co‐localized in the NSCLC cell lines (Figure 6B). Co‐immunoprecipitation was performed to detect the interaction between endogenous KCTD11 and β‐catenin in H1299 cells (Figure 6C). The interaction between exogenous KCTD11 and β‐catenin was also detected by transfection with Myc‐tagged KCTD11 plasmids in A549 cells (Figure 6D). We constructed a KCTD11 mutant plasmid (MYC‐KCTD11‐ΔBTB) and found that MYC‐KCTD11‐ΔBTB could not bind to β‐catenin in A549 cells (Figure 6E, F). Based on the above results, we concluded that KCTD11 interacts with β‐catenin via its own BTB domain.

**TABLE 3:** The relationship between KCTD11expression with β‐catenin membranous expression in 139 cases of NSCLC

	KCTD11		χ^2^	*p*‐value(two sided)
	Positive	Negative		
β‐catenin				
Positive	31	52	4.459	0.035
Negative	32	24		

### 3.8 KCTD11 regulates Hippo pathway activity by β‐catenin

**FIGURE 7:**
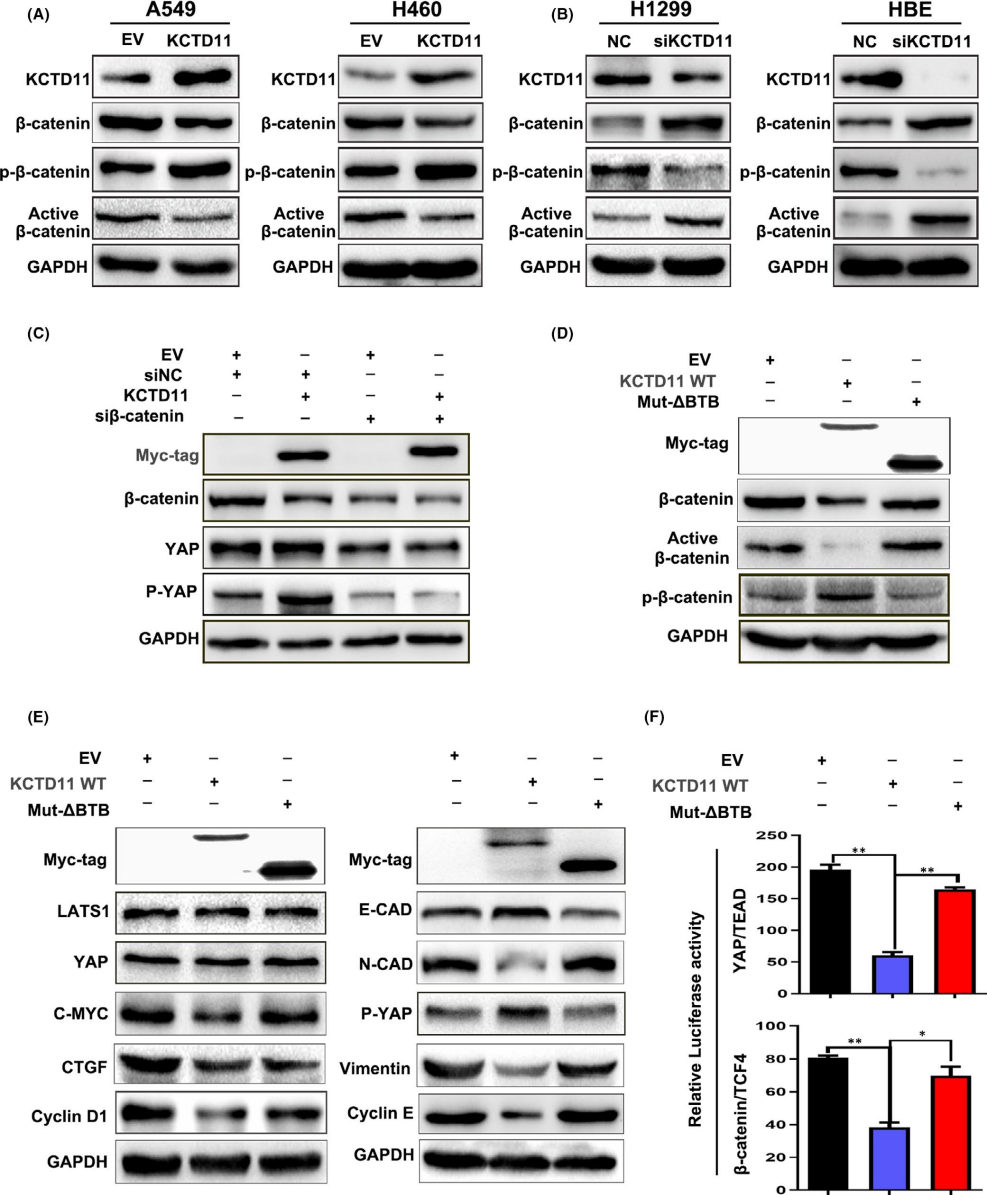
KCTD11 regulates Hippo pathway activity by β‐catenin. (A) Transfection of KCTD11 in A549 and H460 cell lines, Western blot showing that the expression level of active β‐catenin is downregulated and P‐β‐catenin is upregulated. (B) The expression level of active β‐catenin is upregulated, and P‐β‐catenin is downregulated after silencing of KCTD11 in H1299 and HBE cells. (C) KCTD11 inhibits YAP through β‐catenin. A549 cells are transfected with KCTD11 and EV, and si‐catenin and siNC. Western blot showing that after knocking out β‐catenin, the expression levels of YAP and P‐YAP are reduced and that overexpression of KCTD11 could not reverse this change, with the level of YAP and P‐YAP still being inhibited. (D, E) KCTD11 acts as a tumour suppressor through the BTB domain. A549 cells are transfected with EV, KCTD11 and mut‐ΔBTB plasmid and the level of proteins are detected by Western blot. Overexpression of mut‐ΔBTB does not affect the expression of Hippo and wnt pathway‐related proteins and EMT‐related proteins. (F) The Wnt TOPflash reporter and Hippo pGTII luciferase reporter are used to detect the transcriptional activity of YAP / TEAD and β‐catenin / TCF‐4 after transfection of mut‐ΔBTB. Abbreviations: EV, empty vector; siNC, negative control siRNAs

Our experiments showed that KCTD11 negatively regulates the transmission of the Wnt signalling pathway and suppresses the expression of β‐catenin. Further investigation revealed that the levels of active β‐catenin were downregulated, and that P‐β‐catenin was upregulated in KCTD11‐overexpressing A549 and H460 cells, whereas the results were reversed in KCTD11‐depleted H1299 cells and HBE cells (Figure 7A, B). It has been reported that YAP is a direct target gene of Wnt /β‐catenin, and that the deletion of β‐catenin inhibits the expression of YAP [14]. Interestingly, we co‐transfected KCTD11 plasmid and siβ‐catenin in the A549 cell line, but P‐YAP was not upregulated (Figure 7C), indicating that the stimulatory effect on YAP of KCTD11 was dependent on β‐catenin.

To further investigate whether the tumour‐suppressive effect of KCTD11 is mediated by the BTB domain, we transfected a WT KCTD11 plasmid and its mutant MYC‐KCTD11‐ΔBTB in A549 cells. Western blot analysis and dual‐luciferase assay confirmed that the BTB domain is a KCTD11 functional region, which affects the role of KCTD11 in tumour suppression (Figure 7D–F).

## 4 DISCUSSION

Lung cancer, which is one of the leading causes of cancer‐related deaths worldwide, is still on the rise.^25^ Non‐small cell lung cancer accounts for approximately 80% of all lung cancer cases. Although treatment techniques (surgical resection, chemotherapy and radiotherapy) are improving, overall long‐term survival is poor.^26^, ^27^ Thus, finding more effective biomarkers and therapeutic targets for the disease remains essential.

Current research data indicate that KCTD family proteins are involved in different physiological processes of cells and are significantly associated with a variety of diseases.^28^, ^29^, ^30^ However, the expression of KCTD11 in lung cancer and its possible biological effects have not been reported. In this study, we first detected that the expression of KCTD11 in lung cancer specimens was decreased by immunohistochemical methods. The expression of KCTD11 was negatively correlated with the degree of tumour differentiation, high TNM stage and lymph node metastasis in patients with NSCLC. The Kaplan‐Meier database also showed that KCTD11‐positive patients survived significantly longer than KCTD11‐negative patients, suggesting that KCTD11 expression can be considered as a prognostic factor in lung cancer. In vivo and in vitro experiments demonstrated that the overexpression of KCTD11 in lung cancer cell lines significantly inhibited the proliferation and migration of lung cancer cells, with the opposite results seen after knockdown. However, the reason for low KCTD11 expression in non‐small cell lung cancer remains unclear. The possibility of hypermethylation, mutation or enhanced metabolism of the KCTD11 gene in lung cancer cells requires further experimental research and verification.

At present, the research on multiple pathways that regulate tissue growth and tumorigenesis is relatively immature; thus, there is a need to urgently find a target that has a wide range of effects on multiple pathways. In terms of the interaction between the Hippo and Wnt signalling pathways, Hippo pathway inhibition and Wnt pathway activation promote tumorigenesis. Therefore, in order to study the molecular mechanism by which KCTD11 affects the malignant phenotype of lung cancer, we explored the effect of KCTD11 on the expression level of Hippo pathway and Wnt pathway‐related proteins. The results of the dual‐luciferase assay, Western blot and immunofluorescence revealed that KCTD11 significantly enhanced the phosphorylation levels of YAP; inhibited the nuclear translocation of YAP and the transcriptional activity of target genes in the Hippo pathway; significantly reduced β‐catenin expression levels and enhanced β‐catenin phosphorylation levels; inhibited the entry of β‐catenin into the nucleus; and inhibited Wnt pathway activity. Protein‐protein interactions play a vital role in biological functions. The final outcome is that the retention of YAP and β‐catenin in the cytoplasm of the Hippo and Wnt signalling pathways is recognized by E3 ubiquitin ligase β‐trcp, which then leads to their ubiquitination and degradation.^31^, ^32^ The protein KCTD11 contains a BTB domain that can bind to Cullin3 and participate in ubiquitin degradation; nevertheless, the relationship among the three still needs further study.^33^ By co‐immunoprecipitation and immunofluorescence, we found that KCTD11 can bind to β‐catenin and co‐localize in lung cancer tissues and cells. When KCTD11 was transfected and interfered with β‐catenin concurrently, we found that YAP and P‐YAP protein levels decreased with reduced β‐catenin protein levels compared with single KCTD11 transfection. Overall, the present study found that KCTD11 binds to β‐catenin and inhibits β‐catenin nuclear translocation which further inhibits the Hippo pathway, thus leading to decreased proliferation and metastasis of lung cancer cells. In further studies, we directly knocked out the BTB domain and found that KCTD11 cannot bind to β‐catenin, thus losing its role as a tumour suppressor.

## 5 CONCLUSION

The results of this study indicate that the human potassium channel tetramer protein KCTD11 can bind to β‐catenin through its BTB domain in NSCLC cells, inhibit the Wnt pathway, increase Hippo pathway activity, and inhibit the proliferation and migration of lung cancer cells. Therefore, KCTD11 constitutes the link between the Wnt and Hippo pathways, and may be a potential target for lung cancer drug development.

## CONFLICT OF INTEREST

The authors declare that they have no competing interests.

## AUTHOR CONTRIBUTIONS

**Man Yang:** Formal analysis (equal); Methodology (equal); Project administration (equal); Resources (equal); Writing‐original draft (equal). **Ya‐mei Han:** Methodology (equal). **Qiang Han:** Data curation (equal); Funding acquisition (equal). **Xue‐zhu Rong:** Data curation (equal); Software (equal); Validation (equal). **Xiao‐fang Liu:** Formal analysis (equal); Writing‐review & editing (equal). **Xu‐Yong Ln:** Funding acquisition (supporting); Project administration (equal); Writing‐review & editing (equal).

## Supporting information

Fig S1

## Data Availability

The data that support the findings of this study are available from the corresponding author upon reasonable request.

## References

[1] Poon CLC , Liu W , Song Y , et al. A Hippo‐like signaling pathway controls tracheal morphogenesis in drosophila melanogaster. Dev Cell. 2018;47(5):564‐575.e5. 10.1016/j.devcel.2018.09.024 30458981 PMC6281297

[2] Yu FX , Zhao B , Guan KL . Hippo pathway in organ size control, tissue homeostasis, and cancer. Cell. 2015;163(4):811‐828. 10.1016/j.cell.2015.10.044 26544935 PMC4638384

[3] Lin KC , Moroishi T , Meng Z , et al. Regulation of Hippo pathway transcription factor TEAD by p38 MAPK‐induced cytoplasmic translocation. Nat Cell Biol. 2017;19(8):996‐1002. 10.1038/ncb3581 28752853 PMC5541894

[4] Janse van Rensburg HJ , Azad T , Ling M , et al. The Hippo pathway component TAZ promotes immune evasion in human cancer through PD‐L1. Cancer Res. 2018;78(6):1457‐1470. 10.1158/0008-5472.can-17-3139 29339539

[5] Marti P , Stein C , Blumer T , et al. YAP promotes proliferation, chemoresistance, and angiogenesis in human cholangiocarcinoma through TEAD transcription factors. Hepatology. 2015;62(5):1497‐1510. 10.1002/hep.27992 26173433

[6] Martin D , Degese MS , Vitale‐Cross L , et al. Assembly and activation of the Hippo signalome by FAT1 tumor suppressor. Nat Commun. 2018;9(1):2372. 10.1038/s41467-018-04590-1 29985391 PMC6037762

[7] Meliambro K , Wong JS , Ray J , et al. The Hippo pathway regulator KIBRA promotes podocyte injury by inhibiting YAP signaling and disrupting actin cytoskeletal dynamics. J Biol Chem. 2017;292(51):21137‐21148. 10.1074/jbc.M117.819029 28982981 PMC5743086

[8] Konsavage WM Jr , Kyler SL , Rennoll SA , et al. Wnt/β‐catenin signaling regulates Yes‐associated protein (YAP) gene expression in colorectal carcinoma cells. J Biol Chem. 2012;287(15):11730‐11739. 10.1074/jbc.M111.327767 22337891 PMC3320921

[9] Krishnamurthy N , Kurzrock R . Targeting the Wnt/beta‐catenin pathway in cancer: update on effectors and inhibitors. Cancer Treat Rev. 2018;62:50‐60. 10.1016/j.ctrv.2017.11.002 29169144 PMC5745276

[10] Tammela T , Sanchez‐Rivera FJ , Cetinbas NM , et al. A Wnt‐producing niche drives proliferative potential and progression in lung adenocarcinoma. Nature. 2017;545(7654):355‐359. 10.1038/nature22334 28489818 PMC5903678

[11] Emons G , Spitzner M , Reineke S , et al. Chemoradiotherapy resistance in colorectal cancer cells is mediated by Wnt/beta‐catenin signaling. Mol Cancer Res. 2017;15(11):1481‐1490. 10.1158/1541-7786.mcr-17-0205 28811361 PMC5772978

[12] Ji L , Jiang B , Jiang X , et al. The SIAH E3 ubiquitin ligases promote Wnt/beta‐catenin signaling through mediating Wnt‐induced Axin degradation. Genes Dev. 2017;31(9):904‐915. 10.1101/gad.300053.117 28546513 PMC5458757

[13] Deng F , Peng L , Li Z , et al. YAP triggers the Wnt/beta‐catenin signalling pathway and promotes enterocyte self‐renewal, regeneration and tumorigenesis after DSS‐induced injury. Cell Death Dis. 2018;9(2):153. 10.1038/s41419-017-0244-8 29396428 PMC5833613

[14] Imajo M , Miyatake K , Iimura A , et al. A molecular mechanism that links Hippo signalling to the inhibition of Wnt/beta‐catenin signalling. Embo J. 2012;31(5):1109‐1122. 10.1038/emboj.2011.487 22234184 PMC3297994

[15] Park HW , Kim YC , Yu B , et al. Alternative Wnt signaling activates YAP/TAZ. Cell. 2015;162(4):780‐794. 10.1016/j.cell.2015.07.013 26276632 PMC4538707

[16] Azzolin L , Panciera T , Soligo S , et al. YAP/TAZ incorporation in the β‐catenin destruction complex orchestrates the Wnt response. Cell. 2014;158(1):157‐170. 10.1016/j.cell.2014.06.013 24976009

[17] Li X , Chen C , Wang F , et al. KCTD1 suppresses canonical Wnt signaling pathway by enhancing beta‐catenin degradation. PLoS One. 2014;9(4):e94343. 10.1371/journal.pone.0094343 24736394 PMC3988066

[18] Lin CM , Cheng CJ , Yang SS , et al. Generation and analysis of a mouse model of pseudohypoaldosteronism type II caused by KLHL3 mutation in BTB domain. Faseb J. 2019;33(1)**:** 1051‐1061. 10.1096/fj.201801023R 30148674

[19] Gschweitl M , Ulbricht A , Barnes CA , et al. A SPOPL/Cullin‐3 ubiquitin ligase complex regulates endocytic trafficking by targeting EPS15 at endosomes. Elife. 2016;5:e13841. 10.7554/eLife.13841 27008177 PMC4846373

[20] Wang J , Zhu ZH , Yang HB , et al. Cullin 3 targets methionine adenosyltransferase IIalpha for ubiquitylation‐mediated degradation and regulates colorectal cancer cell proliferation. Febs J. 2016;283(13):2390‐2402. 10.1111/febs.13759 27213918

[21] Ji AX , Chu A , Nielsen TK , et al. Structural insights into KCTD protein assembly and cullin3 recognition. J Mol Biol. 2016;428(1):92‐107. 10.1016/j.jmb.2015.08.019 26334369

[22] Pinkas DM , Sanvitale CE , Bufton JC , et al. Structural complexity in the KCTD family of Cullin3‐dependent E3 ubiquitin ligases. Biochem J. 2017;474(22):3747‐3761. 10.1042/bcj20170527 28963344 PMC5664961

[23] Travis WD , Brambilla E , Burke AP , et al. Introduction to the 2015 World Health Organization classification of tumors of the lung, pleura, thymus, and heart. J Thorac Oncol. 2015;10(9):1240‐1242. 10.1097/jto.0000000000000663 26291007

[24] Lin XY , Zhang XP , Wu JH , et al. Expression of LATS1 contributes to good prognosis and can negatively regulate YAP oncoprotein in non‐small‐cell lung cancer. Tumour Biol. 2014;35(7):6435‐6443. 10.1007/s13277-014-1826-z 24682895

[25] Zhang Y , Ren JS , Huang HY , et al. International trends in lung cancer incidence from 1973 to 2007. Cancer Med. 2018;7(4):1479‐1489. 10.1002/cam4.1359 29542259 PMC5911623

[26] Bagcchi S . Lung cancer survival only increases by a small amount despite recent treatment advances. Lancet Respir Med. 2017;5(3):169. 10.1016/s2213-2600(17)30041-3 28169198

[27] Detterbeck FC , Boffa DJ , Kim AW , Tanoue LT . The eighth edition lung cancer stage classification. Chest. 2017;151(1):193‐203. 10.1016/j.chest.2016.10.010 27780786

[28] Liu Z , Song F , Ma ZL , et al. Bivalent copper ions promote fibrillar aggregation of KCTD1 and induce cytotoxicity. Sci Rep. 2016;6(1):32658. 10.1038/srep32658 27596723 PMC5011690

[29] Zuo H , Glaaser I , Zhao Y , et al. Structural basis for auxiliary subunit KCTD16 regulation of the GABAB receptor. Proc Natl Acad Sci U S A. 2019;116(17):8370‐8379. 10.1073/pnas.1903024116 30971491 PMC6486783

[30] Metz KA , Teng X , Coppens I , et al. KCTD7 deficiency defines a distinct neurodegenerative disorder with a conserved autophagy‐lysosome defect. Ann Neurol. 2018;84(5):766‐780. 10.1002/ana.25351 30295347 PMC6295419

[31] Zhao B , Li L , Tumaneng K , et al. A coordinated phosphorylation by Lats and CK1 regulates YAP stability through SCF(beta‐TRCP). Genes Dev. 2010;24(1)**:** 72‐85. 10.1101/gad.1843810 20048001 PMC2802193

[32] Simonetta KR , Taygerly J , Boyle K , et al. Prospective discovery of small molecule enhancers of an E3 ligase‐substrate interaction. Nat Commun. 2019;10(1):1402. 10.1038/s41467-019-09358-9 30926793 PMC6441019

[33] Zheng S , Abreu N , Levitz J , Kruse AC . Structural basis for KCTD‐mediated rapid desensitization of GABAB signalling. Nature. 2019;567(7746)**:** 127‐131. 10.1038/s41586-019-0990-0 30814734 PMC6405316

