# Supplementary figures and images for: KCTD11 inhibits progression of lung cancer by binding to β‐catenin to regulate the activity of the Wnt and Hippo pathways
Source: J Cell Mol Med. 2021 Aug 28;25(19):9411–26. doi: 10.1111/jcmm.16883 (PMC8500973; doi:10.1111/jcmm.16883)

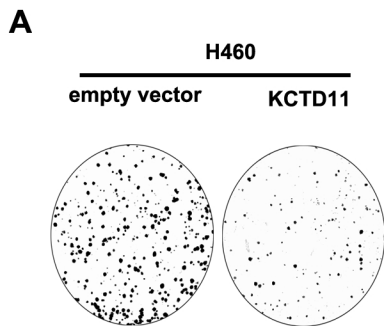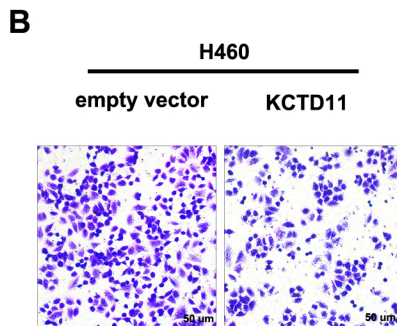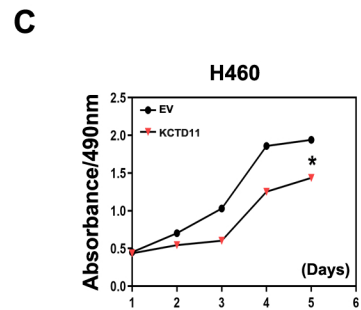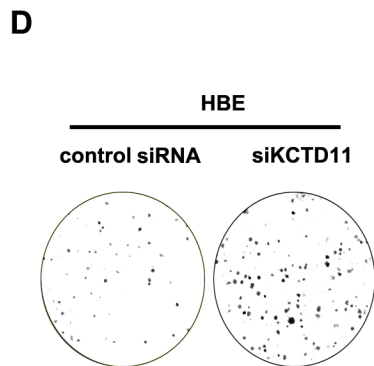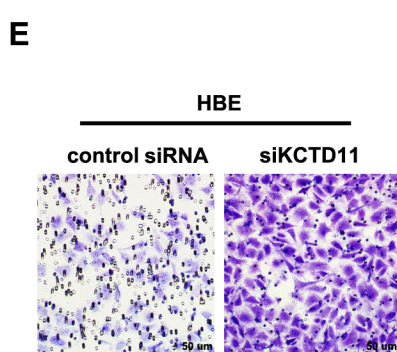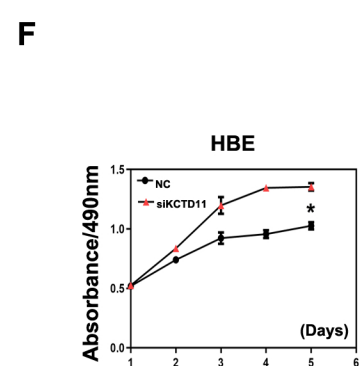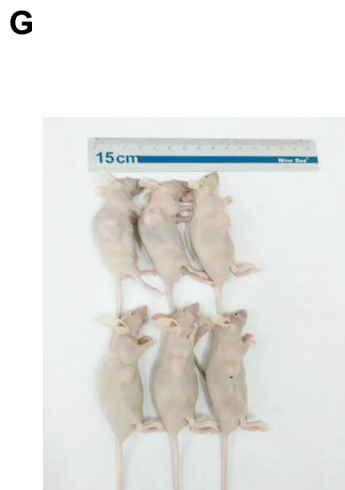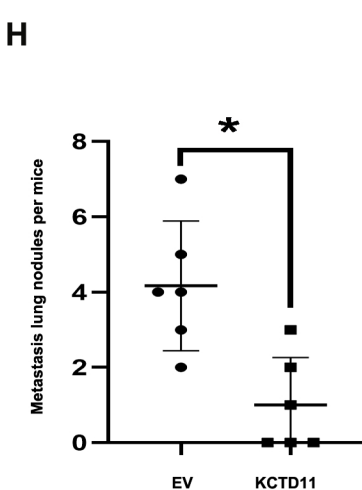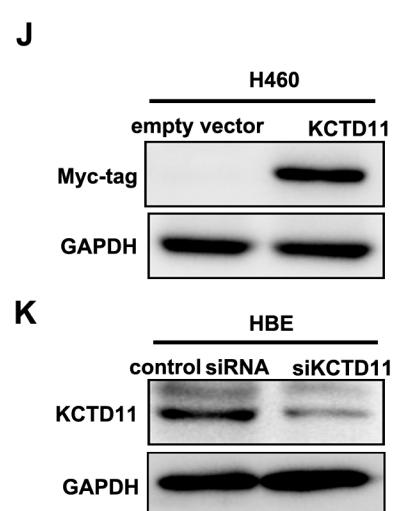

Supplement: Supplementary file 1 — Fig S1 [file JCMM-25-9411-s001.pdf]
